# From Molecular Genetics to Phylodynamics: Evolutionary Relevance of Mutation Rates Across Viruses

**DOI:** 10.1371/journal.ppat.1002685

**Published:** 2012-05-03

**Authors:** Rafael Sanjuán

**Affiliations:** Institut Cavanilles de Biodiversitat i Biologia Evolutiva, Universitat de València, Paterna, Valencia, Spain; North Carolina State University, United States of America

## Abstract

Although evolution is a multifactorial process, theory posits that the speed of molecular evolution should be directly determined by the rate at which spontaneous mutations appear. To what extent these two biochemical and population-scale processes are related in nature, however, is largely unknown. Viruses are an ideal system for addressing this question because their evolution is fast enough to be observed in real time, and experimentally-determined mutation rates are abundant. This article provides statistically supported evidence that the mutation rate determines molecular evolution across all types of viruses. Properties of the viral genome such as its size and chemical composition are identified as major determinants of these rates. Furthermore, a quantitative analysis reveals that, as expected, evolution rates increase linearly with mutation rates for slowly mutating viruses. However, this relationship plateaus for fast mutating viruses. A model is proposed in which deleterious mutations impose an evolutionary speed limit and set an extinction threshold in nature. The model is consistent with data from replication kinetics, selection strength and chemical mutagenesis studies.

## Author Summary

Viruses are an excellent system for addressing the evolutionary implications of mutation because their mutation rates vary by orders of magnitude, and their evolution takes place within the time frame of human observation. Theory posits a direct relationship between these two processes, but this has rarely been tested empirically. This work shows that evolution rates in nature correlate with experimentally-determined mutation rates for the major viral groups, and identifies key genome properties determining these rates. Current theory allows us to predict evolution rates accurately for slowly-mutating viruses but fails for the fastest mutating viruses. To solve this limitation, a model in which deleterious mutations play a key evolutionary role is proposed.

## Introduction

Mutations result from biochemical processes such as replication errors, editing, or nucleic acid damage, but their spread and fixation is a population-genetics process that takes place over much broader scales. Remarkably, the neutral theory of molecular evolution posits that the mutation rate should be the sole determinant of molecular evolution rates [1], and adaptation theory also assigns a central role to mutation [2], [3]. However, it is unclear to what extent this direct association between mutation and evolution holds true in nature because a variety of complex selective, ecological and demographical factors can potentially affect the evolutionary process at the molecular level [2], [4], [5]. Viruses offer an excellent system for addressing this question because their evolution is fast enough to be measured directly from isolates collected within timescales of years [6] and their mutation rates vary by several orders of magnitude [7]. The viral mutation rate has been shown to determine pathogenesis [8], [9], the risk of drug resistance [10], vaccine efficacy [11], [12], the success of antiviral treatments [13]–[15], or the likelihood of emergence of new diseases [16], [17]. It is also known that most RNA viruses evolve extremely fast owing to their high mutation rates, which in turn are explained biochemically by the absence of proofreading or repair mechanisms [5], [15]. However, our current knowledge of the evolutionary consequences of viral mutation is mainly qualitative or restricted to a small subset of viruses.

By undertaking a systematic quantitative analysis, this work shows that the evolution rates of major viral groups in nature are consistent with mutation rate estimates obtained under controlled laboratory conditions. The size, polarity and number of genome strands are identified as major determinants of viral mutation and evolution. According to a purely neutral model, the evolution rate should increase linearly with the mutation rate, and this prediction is confirmed for viruses with relatively low mutation rates. However, evolution rates increase less than linearly as mutation rates become higher. A model in which the fitness load imposed by transient deleterious mutations retards molecular evolution is proposed, and the inferred parameters are tested using data from site-directed mutagenesis studies and other sources of evidence. This model predicts that further increases of the mutation rate would have a negative impact on viral evolution and suggests that RNA viruses replicate near an extinction threshold in nature.

## Results/Discussion

### Variation in mutation and evolution rates across viruses

**Figure 1:**
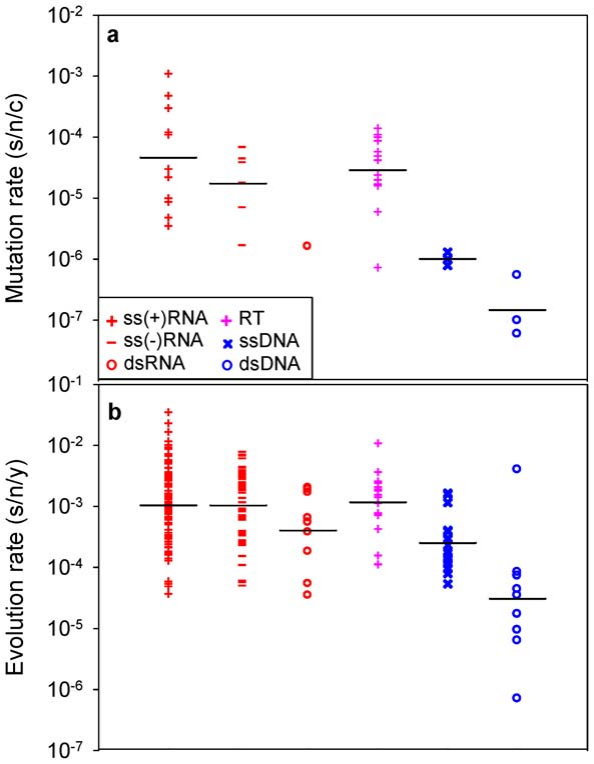
Mutation and evolution rate estimates for the major groups defined by the Baltimore classification of viruses. **a**: mutation rates; **b**: evolution rates. Each data point corresponds to an individual estimate. Bars indicate log-scale (geometric) means.

**Figure 2:**
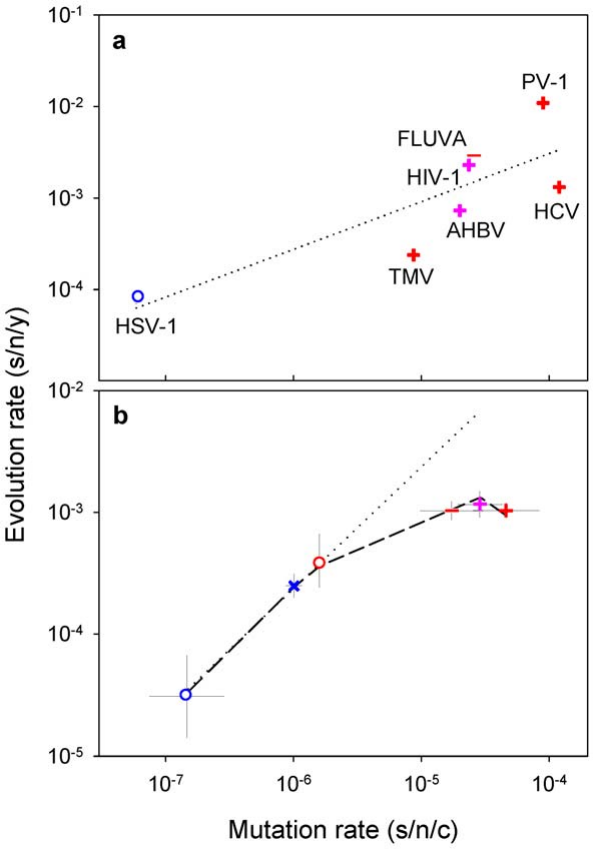
Relationship between mutation and evolution rates across viruses. Symbols for each Baltimore group are the same as in Figure 1. **a**: evolution rates versus mutation rates for seven viruses (HSV-1: herpes simplex virus 1; TMV: tobacco mosaic virus; AHBV: avian hepatitis B virus; FLUVA: influenza A virus; HIV-1: human immunodeficiency virus 1; PV-1: poliovirus 1; HCV: hepatitis C virus). **b**: log-scale mean ± SEM mutation and evolution rates for each Baltimore group. The dotted line indicates the prediction from a purely neutral model, whereas the dashed line corresponds to a model that incorporates deleterious mutations.

A recent compilation of experimentally-determined mutation rates yielded 37 standardized estimates for 23 viruses [7]. These rates range from 10^−8^ to 10^−3^ substitutions per nucleotide site per cell infection (s/n/c) and vary significantly among the major groups defined by the Baltimore classification of viruses (Figure 1a; nested ANOVA: *P* = 0.002). For evolution rates, 223 estimates corresponding to 84 different viruses were collected (Text S1), all of which were obtained using Bayesian analysis of dated sequences [18] and after validation of the molecular clock. This methodological consistency is critical to make reliable comparisons since evolution rates can vary strongly depending on the estimation procedure [19], [20]. The collected evolution rates range from 10^−6^ to 10^−2^ substitutions per nucleotide site per year (s/n/y) and also vary significantly among Baltimore groups (Figure 1b; nested ANOVA: *P*<0.001). The fastest evolution corresponds to single-stranded (ss) RNA and reverse-transcribing (RT) viruses, followed by double stranded (ds) RNA and ssDNA viruses, whereas dsDNA viruses evolve more slowly on average (Tukey's post-hoc test: *P*<0.05). This confirms the well-known difference between RNA and DNA viruses [5], [15] and, further, demonstrates that single-stranded viruses tend to evolve faster than double-stranded viruses regardless of whether their genetic material is RNA or DNA (two-way nested ANOVA excluding RT viruses: *P*<0.001). Among the seven viruses for which both mutation and evolution rates have been determined, these correlate positively (Figure 2a; Pearson *r* = 0.813, *P* = 0.026). Furthermore, when averages are calculated for each Baltimore group using all available estimates, mutation and evolution rates show a strongly positive correlation (Figure 2b; *r* = 0.946, *P* = 0.004).

### Evolutionary basis for the correlation between mutation and evolution rates

Consider first a purely neutral model in which the evolution rate *K* is proportional to the mutation rate *μ* [1]. Therefore, 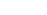, or equivalently, 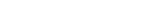, log-scales being here more appropriate for model fitting because the data range several orders of magnitude. Notice that the model specifically predicts that the linear relationship between log *K* and log *μ* has slope 1.0. The value of *a* depends on the number of cell infection cycles (generations) per year (*g*) and on the fraction of effectively neutral mutations (*α*) such that 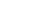. For viruses with relatively low mutation rates (dsDNA, ssDNA and dsRNA viruses), this model fits the data accurately (*r^2^* = 0.995, Figure 2b), yielding log_10_ *a* = 2.37±0.02 (SEM). This is in full agreement with the neutral theory, although adaptive evolution may produce a similar pattern in some cases [2]. However, *K* increases less than linearly with *μ* for the fastest mutating viruses (ssRNA an RT viruses) and the overall fit of the above model is poor (*r^2^* = 0.432). Because transient deleterious mutations are highly abundant in RNA virus populations [21], the spread and fixation of mutations should be slowed down by the presence of deleterious mutations elsewhere in the genome. Specifically, the expected fraction of individuals not carrying these mutations is 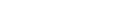, where *G* is genome size and *s_H_* the harmonic mean of selection coefficients [3]. Taking this into account, the predicted evolution rate becomes 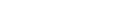, with 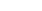 and 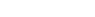. This modification strongly improves the model (Figure 2b; *r^2^* = 0.995; partial *F*-test: *P*<0.001), yielding log_10_ *a* = 2.387±0.027 and *b* = 3.744±0.172. Therefore, short-lived deleterious mutations appear to play a key role is setting the rate of molecular evolution in RNA viruses. Although this effect concerns mainly neutral evolution, it should also be relevant to models of adaptation [3].

The inferred values for parameters *a* and *b* are consistent with independent sources of evidence. Site-directed mutagenesis studies in which the fitness effects of point mutations were determined for tobacco etch virus [22], bacteriophage Qβ [23] and vesicular stomatitis virus [24] (three ssRNA viruses) gave *α*≈0.27 and *s_H_* values ranging from 0.172 to 0.338 (Text S1). Using 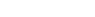 allows us to obtain an estimate of *b* which ranges from 1.475 to 5.177 and includes the value *b* = 3.744 inferred above. However, the interval is relatively wide and thus does not provide a very stringent test of the model. Additionally, although the three viruses belong to different families and infect widely different hosts, some caution is granted because the estimated *s_H_* was based on three species only. Concerning parameter *a*, if we again assume *α* = 0.27 the estimated number of cell infection cycles per year averaged across viruses is 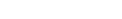. This is equivalent to one cell infection every 10 h, which is a realistic value for a variety of actively replicating eukaryotic viruses [25]–[28]. Therefore, the above model linking mutation and evolution rates is in broad agreement with empirical evidence from quantitative replication kinetics, selection strength and chemical mutagenesis studies.

### An extinction threshold in nature

**Figure 3:**
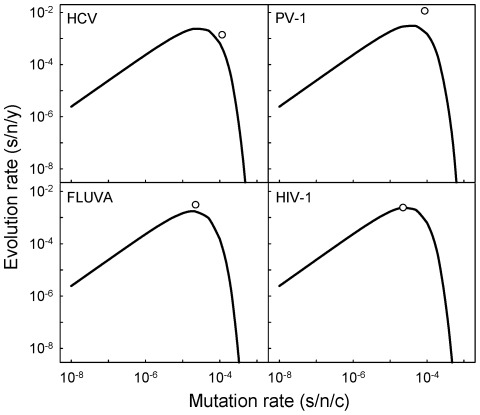
Expected relationship between mutation and evolution rates according to the neutral-deleterious evolution model for four human viruses: HCV (hepatitis C virus), PV-1 (poliovirus 1), FLUVA (influenza A virus), and HIV-1 (human immunodeficiency virus 1). Curves indicate the prediction obtained using log_10_ *a* = 2.387, *b* = 3.744, and the corresponding genome size of each virus. White dots show the observed average rates. These four viruses were chosen for representation because of the relatively high number of estimates available (see [7] and Text S1). Fewer data are available for the other three viruses appearing in Figure 2a, and their predicted rates deviated more from the observed values.

Previous experimental work has shown that slight elevations of the mutation rate (on the order of threefold) can lead to drastic fitness losses in a variety of ssRNA and RT viruses and often achieve mutagenesis-induced population extinction in the laboratory, suggesting a possible antiviral strategy [13]–[15]. However, evidence showing the relevance of these observations in natural populations has remained elusive. The above model predicts that the rate of evolution should be maximal when the genomic mutation rate is 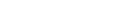 (i.e. when 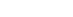), and then decays exponentially. The mean genomic mutation rates of ss(+)RNA viruses (0.663±0.417), ss(−)RNA viruses (0.372±0.124) and RT viruses (0.445±0.116) are slightly higher but not significantly different from this value (one-sample *t*-tests: *P*≥0.150), implying that these viruses replicate close to the optimal mutation rate. However, this also means that further increases of the mutation rate would actually reduce the evolution rates of these viruses in nature and potentially endanger their survival. For instance, on average, a threefold increase in the mutation rate of ss(+)RNA viruses would produce a 48-fold evolutionary slowdown. Figure 3 shows the predicted relationship between mutation and evolution rates for hepatitis C virus, poliovirus 1, influenza A virus, and human immunodeficiency virus 1, four well-studied human viruses.

### Correlation between genome size, mutation rate, and evolution rate

**Figure 4:**
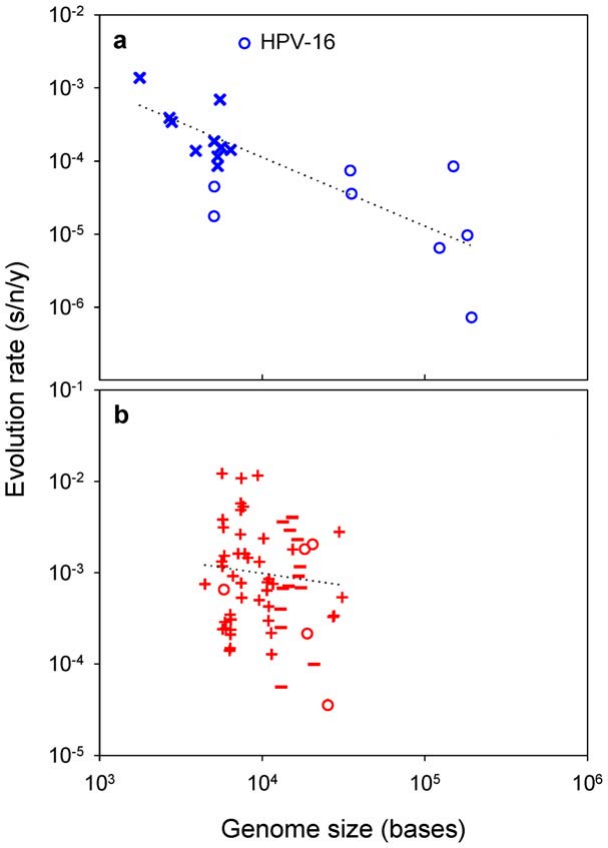
Viral evolution rate versus genome size. **a**: DNA viruses; **b**: RNA viruses. The least-squares linear regression lines are shown. Symbols for each Baltimore group are the same as in Figure 1.

Drake's rule establishes that the genomic mutation rate is approximately constant across DNA microorganisms (including viruses) and equal to 0.003 substitutions per generation [29], [30]. Since *μ*≈0.003/*G*, it is possible to use *G* as an inverse correlate of *μ* to further test the association between mutation and evolution rates. If mutation rates determine evolution, DNA viruses with small genomes should tend to evolve faster than those with large genomes. Confirming this prediction, the evolution rates of 19 different DNA viruses correlate negatively with their genome sizes (Figure 4a; partial *r* = −0.707, *P* = 0.001) and, importantly, this correlation remains significant after accounting for the fact that ssDNA viruses usually have smaller genomes than dsDNA viruses (partial *r* = −0.551, *P* = 0.022). A linear regression of the form 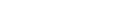 gives *m* = −0.906±0.216 and *p* = −0.349±0.890. The estimate of *m* does not deviate significantly from 1.0 (*t*-test: *P* = 0.667), further supporting the linear relationship between *K* and *μ* shown above for slowly-mutating viruses. An apparent outlier is the human papillomavirus (HPV) 16, which evolves faster than expected from its genome size. However, this rate was obtained from sequences sampled only three years apart, and there is a known tendency for evolution rate estimates to become inflated in the short-term [20]. Indeed, the HPV-16 estimate and those for varicella zoster and human adenovirus C are considered unreliable [31]. Small dsDNA viruses (HPV-16 and two polyomaviruses) are also problematic because their mutation rates have not been determined and there is little consensus about their evolution rates [31]. However, supporting the robustness of the results, the above correlations remain unaffected or even improve after removing these five viruses (*r* = −0.791, *P* = 0.001 and *r* = −0.629, *P* = 0.029, respectively).

Concerning RNA viruses, recent work suggests that there may also be an inverse relationship between mutation rates and genome sizes, although less evident than for DNA viruses [7] probably because their narrower genome size range makes it more difficult to demonstrate this association. Interestingly, genome sizes and evolution rates also correlate negatively among RNA viruses [32], yet this correlation is much weaker than for DNA viruses (Figure 4b; partial correlation excluding RT viruses: *r* = −0.267, *P* = 0.038).

### Other factors determining viral evolution

Several factors can determine viral evolution in addition to the mutation rate. For instance, viruses undergoing fewer replication cycles per time unit should evolve more slowly, and this appears to be the case of water- or vector-borne viruses which spend longer periods of inactivity than directly transmitted viruses [32], [33]. In latently integrated retroviruses, viral replication is carried out by the host machinery, thus reducing dramatically the mutation rate compared to actively replicating viruses. This can explain why a very low rate of evolution was inferred for foamy virus based on a well-supported host-virus co-speciation pattern [34]. Similar results have been obtained for vertically transmitted human T-cell leukemia viruses [35] and for papilloma viruses coevolving with felids [36]. On the other hand, positive selection associated with recent host jumps or immune pressure can accelerate evolution, and a similar effect occurs in viruses experiencing strong transmission bottlenecks because this reduces the effective population size and relaxes selection against deleterious mutations [17]. Taking these factors into account and given that estimation errors are usually large, it is not surprising that mutation and evolution rates show considerable scatter, and that their relationship becomes evident only after averaging large and comparable datasets.

Another necessary caveat is that time-structured sequence data spanning years or decades often contain short-lived polymorphisms. Among other factors, this explains why evolution rates inferred in this manner are generally higher than those based on long-term calibration points such as co-speciation events [20], and warns against comparing rates obtained by such different methods. Although the dataset used here was based on dated samples only and was methodologically consistent, sampling timespans were inevitably variable, but this was accounted for in the statistical analysis. It was not possible to use studies based on co-speciation events for testing the model because estimates obtained in this way are not reliable for most viral types. Finally, a potential methodological pitfall is that some viral species and families have been more extensively studied than others, thus introducing sampling bias. A more robust analysis consists of giving the same weight to all species and families, independent of the number of estimates available for each. This alternative averaging method gave very similar results (see Methods for details).

### Conclusion

The classical notion that RNA viruses are the fastest mutating and evolving entities in nature has been revised, after several recent reports showing that the evolution rates of ssDNA viruses are similarly high [5]. This is compatible with the finding that mutation and evolution rates are generally higher in single-stranded viruses than in double-stranded viruses (RNA or DNA), a possible explanation being the greater instability of single-stranded nucleic acids [37]. On the other hand, mutation and evolution rates appear to vary smoothly across viruses and, therefore, defining discrete categories may not be a helpful approach. From a broader perspective, despite the extreme diversity of viral types, the above model provides a simple and general framework for how mutation rates determine viral evolution. This generality is achieved after incorporating the impact of deleterious mutations on evolution, which should be particularly significant in ssRNA and RT viruses. The proposed model is consistent with several independent sources of evidence but, to further consolidate it, additional empirical studies will be needed. For instance, selection strength data have been obtained experimentally only for a handful of viruses. Also, the mutation rates in eukaryotic ssDNA and dsRNA viruses are largely unknown, and a similar uncertainty exists for evolution rates in bacteriophages and small dsDNA viruses. The present work provides well-defined predictions for addressing these issues in the future.

## Methods

Mutation rates were taken directly from a recent meta-analysis [7]. Evolution rates were selected from the literature according to the inclusion criteria indicated in the text. For methodological consistency, estimates based on long-term virus-host co-speciation events were not used. Although co-speciation is well supported for some DNA viruses, it is not for most RNA viruses [38]. Since evolution rates are known to be time-dependent [20], inclusion of co-speciation data for DNA viruses would inflate differences between DNA and RNA viruses. Similarly, within and among host evolution rates differ systematically [39] and thus the former were not used. Estimates from the same study but corresponding to different datasets (genes or groups of sequences) were treated as independent observations, whereas those obtained from the same dataset using different methods were averaged before analysis or the best-fit value was used if available. Because raw rates ranged several orders of magnitude log-transformed data were used. Data normality was satisfied within each Baltimore category after this transformation for both mutation and evolution rates (Kolmogorov-Smirnov tests: *P*≥0.258).

In ANOVA and correlation tests, the sampling timespan was used as a covariate to account for evolution rate time-dependency. Mean rates for each Baltimore category were calculated directly from individual observations. Although the Baltimore classification distinguishes between RT-DNA and RT-RNA viruses, these two groups were pooled because there were data for few species, but similar results were obtained using the full set of Baltimore groups (not shown). For model fitting, log-scale means were also used except for the *μG* term appearing in equation 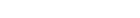 for which arithmetic means were used. For the ss(+)RNA group this mean (1.185±0.478) was strongly affected by a few extremely high estimates and thus, a previously-defined subset of more reliable estimates [7] was used instead. However, using *μG* = 1.185, the model with deleterious mutations also provided a significant improvement over the purely neutral model (partial *F*-test: *P* = 0.017), yielding log_10_ *a* = 2.287±0.105, *b* = 2.116±0.449, and *r* ^2^ = 0.914. To account for the fact that some viral species or families were more represented than others, the analysis was repeated after calculating unweighted averages hierarchically first for each species, then for each family, then for each Baltimore group. In addition to reducing bias, this method accounts for phylogenetic relatedness (on the other hand, it increases error because of the fewer estimates available for some groups). The correlation between mutation and evolution rates was maintained (*r* = 0.923, *P* = 0.009) and the model with deleterious mutations provided again the best fit (*r* ^2^ = 0.961; partial *F*-test: *P*<0.001). To further test the robustness of the results, the analysis was also redone using medians instead of means. The correlation between mutation and evolution rates was *r* = 0.956 (*P* = 0.003) and, again, the model with deleterious mutations explained the data better than the purely neutral one (*r* ^2^ = 0.926; partial *F*-test: *P* = 0.012). In these two alternative analyses, the average *μG* for ss(+)RNA viruses was also calculated using the subset of more reliable estimates.

## Supporting Information

Text S1 Original datasets and references. **Table S1:** Evolution rate dataset. For each estimate, the viral species, family and Baltimore group, as well as the genome size and the time span of the study are indicated. **Table S2.** Fitness effects of points mutations determined by site-directed mutagenesis. A list of mutated sites, relative fitness values and per-generation selection coefficients is shown for tobacco etch virus, bacteriophage Qβ, and vesicular stomatitis virus.
(PDF)

## References

[1] Kimura M (1983). The neutral theory of molecular evolution.

[2] Grenfell BT, Pybus OG, Gog JR, Wood JL, Daly JM (2004). Unifying the epidemiological and evolutionary dynamics of pathogens.. Science.

[3] Orr HA (2000). The rate of adaptation in asexuals.. Genetics.

[4] Bromham L (2011). The genome as a life-history character: why rate of molecular evolution varies between mammal species.. Philos Trans R Soc Lond B Biol Sci.

[5] Duffy S, Shackelton LA, Holmes EC (2008). Rates of evolutionary change in viruses: patterns and determinants.. Nat Rev Genet.

[6] Drummond A, Pybus OG, Rambaut A, Forsberg R, Rodrigo AG (2003). Measurably evolving populations.. Trends Ecol Evol.

[7] Sanjuán R, Nebot MR, Chirico N, Mansky LM, Belshaw R (2010). Viral mutation rates.. J Virol.

[8] Pfeiffer JK, Kirkegaard K (2005). Increased fidelity reduces poliovirus fitness and virulence under selective pressure in mice.. PLoS Pathog.

[9] Vignuzzi M, Stone JK, Arnold JJ, Cameron CE, Andino R (2006). Quasispecies diversity determines pathogenesis through cooperative interactions in a viral population.. Nature.

[10] Perelson AS (2002). Modelling viral and immune system dynamics.. Nat Rev Immunol.

[11] Davenport MP, Loh L, Petravic J, Kent SJ (2008). Rates of HIV immune escape and reversion: implications for vaccination.. Trends Microbiol.

[12] Vignuzzi M, Wendt E, Andino R (2008). Engineering attenuated virus vaccines by controlling replication fidelity.. Nat Med.

[13] Anderson JP, Daifuku R, Loeb LA (2004). Viral error catastrophe by mutagenic nucleosides.. Annu Rev Microbiol.

[14] Bull JJ, Sanjuán R, Wilke CO (2007). Theory of lethal mutagenesis for viruses.. J Virol.

[15] Domingo E (2006). Quasispecies: concept and implications for virology.

[16] Holmes EC (2009). The evolution and emergence of RNA viruses.

[17] Pepin KM, Lass S, Pulliam JR, Read AF, Lloyd-Smith JO (2010). Identifying genetic markers of adaptation for surveillance of viral host jumps.. Nat Rev Microbiol.

[18] Drummond AJ, Rambaut A (2007). BEAST: Bayesian evolutionary analysis by sampling trees.. BMC Evol Biol.

[19] Holmes EC (2003). Molecular clocks and the puzzle of RNA virus origins.. J Virol.

[20] Ho SY, Lanfear R, Bromham L, Phillips MJ, Soubrier J (2011). Time-dependent rates of molecular evolution.. Mol Ecol.

[21] Pybus OG, Rambaut A, Belshaw R, Freckleton RP, Drummond AJ (2007). Phylogenetic evidence for deleterious mutation load in RNA viruses and its contribution to viral evolution.. Mol Biol Evol.

[22] Carrasco P, de la Iglesia F, Elena SF (2007). Distribution of fitness and virulence effects caused by single-nucleotide substitutions in Tobacco Etch virus.. J Virol.

[23] Domingo-Calap P, Cuevas JM, Sanjuán R (2009). The fitness effects of random mutations in single-stranded DNA and RNA bacteriophages.. PLoS Genet.

[24] Sanjuán R, Moya A, Elena SF (2004). The distribution of fitness effects caused by single-nucleotide substitutions in an RNA virus.. Proc Natl Acad Sci U S A.

[25] Althaus CL, De Vos AS, de Boer RJ (2009). Reassessing the human immunodeficiency virus type 1 life cycle through age-structured modeling: life span of infected cells, viral generation time, and basic reproductive number, R0.. J Virol.

[26] Cuevas JM, Moya A, Sanjuán R (2005). Following the very initial growth of biological RNA viral clones.. J Gen Virol.

[27] Malpica JM, Fraile A, Moreno I, Obies CI, Drake JW (2002). The rate and character of spontaneous mutation in an RNA virus.. Genetics.

[28] Nobusawa E, Sato K (2006). Comparison of the mutation rates of human influenza A and B viruses.. J Virol.

[29] Drake JW (1991). A constant rate of spontaneous mutation in DNA-based microbes.. Proc Natl Acad Sci U S A.

[30] Lynch M (2010). Evolution of the mutation rate.. Trends Genet.

[31] Firth C, Kitchen A, Shapiro B, Suchard MA, Holmes EC (2010). Using time-structured data to estimate evolutionary rates of double-stranded DNA Viruses.. Mol Biol Evol.

[32] Jenkins GM, Rambaut A, Pybus OG, Holmes EC (2002). Rates of molecular evolution in RNA viruses: a quantitative phylogenetic analysis.. J Mol Evol.

[33] Hanada K, Suzuki Y, Gojobori T (2004). A large variation in the rates of synonymous substitution for RNA viruses and its relationship to a diversity of viral infection and transmission modes.. Mol Biol Evol.

[34] Switzer WM, Salemi M, Shanmugam V, Gao F, Cong ME (2005). Ancient co-speciation of simian foamy viruses and primates.. Nature.

[35] Salemi M, Lewis M, Egan JF, Hall WW, Desmyter J (1999). Different population dynamics of human T cell lymphotropic virus type II in intravenous drug users compared with endemically infected tribes.. Proc Natl Acad Sci U S A.

[36] Rector A, Lemey P, Tachezy R, Mostmans S, Ghim SJ (2007). Ancient papillomavirus-host co-speciation in Felidae.. Genome Biol.

[37] Frederico LA, Kunkel TA, Shaw BR (1990). A sensitive genetic assay for the detection of cytosine deamination: determination of rate constants and the activation energy.. Biochemistry.

[38] Holmes EC (2008). Evolutionary history and phylogeography of human viruses.. Annu Rev Microbiol.

[39] Pybus OG, Rambaut A (2009). Evolutionary analysis of the dynamics of viral infectious disease.. Nat Rev Genet.

