# Supplementary material for: From Molecular Genetics to Phylodynamics: Evolutionary Relevance of Mutation Rates Across Viruses
Source: PLoS Pathog. 2012 May 3;8(5):e1002685. doi: 10.1371/journal.ppat.1002685 (PMC3342999; doi:10.1371/journal.ppat.1002685)
Supplement: Text S1 — Original datasets and references. Table S1: Evolution rate dataset. For each estimate, the viral species, family and Baltimore group, as well as the genome size and the time span of the study are indicated. Table S2. Fitness effects of points mutations determined by site-directed mutagenesis. A list of mutated sites, relative fitness values and per-generation selection coefficients is shown for tobacco etch virus, bacteriophage Qβ, and vesicular stomatitis virus. (PDF) [file ppat.1002685.s001.pdf]

Table S1: evolution rate dataset

Group	Family	Virus	G^*	Evolution rate (K)[†]	Time_span	Ref.
ss(+)RNA	Arteriviridae	Porcine reproductive and respiratory syndrome virus	15428	1.80E-03	2	[1]
ss(+)RNA	Calciviridae	Norwalk virus	7654	4.97E-03 (4.30E-03, 5.60E-03, 5.10E-03)	34	[2]
ss(+)RNA	Calciviridae	Norwalk virus	7654	5.63E-03 (4.16E-03, 7.39E-03, 5.80E-03)	31	[3]
ss(+)RNA	Calciviridae	Rabbit hemorrhagic disease virus	7437	7.70E-04	53	[4]
ss(+)RNA	Coronaviridae	Avian coronavirus	27608	9.40E-04	13	[5]
ss(+)RNA	Coronaviridae	Avian coronavirus	27608	1.70E-04	25	[5]
ss(+)RNA	Coronaviridae	Avian coronavirus	27608	2.40E-04	41	[5]
ss(+)RNA	Coronaviridae	Bovine coronavirus	31028	5.37E-04	33	[6]
ss(+)RNA	Coronaviridae	Human coronavirus 229E	27317	3.28E-04	33	[7]
ss(+)RNA	Coronaviridae	SARS coronavirus	29749	2.79E-03	2	[8]
ss(+)RNA	Coronaviridae	SARS coronavirus	29749	2.82E-03	4	[9]
ss(+)RNA	Dicistroviridae	Taura syndrome virus	10205	2.37E-03	16	[10]
ss(+)RNA	Flaviviridae	Dengue virus	10735	6.50E-04	43	[11]
ss(+)RNA	Flaviviridae	Dengue virus	10735	1.07E-03	26	[12]
ss(+)RNA	Flaviviridae	Dengue virus	10735	6.50E-04	51	[13]
ss(+)RNA	Flaviviridae	Dengue virus	10735	8.48E-04	7	[14]
ss(+)RNA	Flaviviridae	GB-C virus	9392	3.42E-03 (4.13E-03, 4.15E-03, 2.10E-03, 4.45E-03, 4.13E-03, 2.09E-03, 5.03E-03, 4.99E-03, 1.85E-03)	9	[15]
ss(+)RNA	Flaviviridae	GB-C virus	9392	3.50E-02 (5.43E-02, 4.33E-02, 3.28E-02, 1.94E-02, 3.95E-02, 3.95E-02, 2.49E-02, 4.30E-02, 3.16E-02)	4	[15]
ss(+)RNA	Flaviviridae	GB-C virus	9392	9.33E-03 (2.19E-02, 1.46E-02, 3.53E-03, 2.74E-02, 7.92E-03, 2.61E-03, 2.89E-02, 8.67E-03, 3.34E-03)	3	[15]
ss(+)RNA	Flaviviridae	GB-C virus	9392	1.65E-02 (3.55E-02, 2.12E-02, 1.52E-02, 3.58E-02, 8.49E-03, 6.99E-03, 3.24E-02, 1.14E-02, 1.02E-02)	4	[15]
ss(+)RNA	Flaviviridae	Hepatitis C virus	9628	1.39E-03	19	[16]
ss(+)RNA	Flaviviridae	Hepatitis C virus	9628	1.15E-03	19	[16]
ss(+)RNA	Flaviviridae	Hepatitis C virus	9628	1.30E-03	30	[17]
ss(+)RNA	Flaviviridae	Hepatitis C virus	9628	1.00E-03	30	[17]
ss(+)RNA	Flaviviridae	Hepatitis C virus	9628	2.10E-03	17	[17]
ss(+)RNA	Flaviviridae	Hepatitis C virus	9628	1.20E-03	17	[17]
ss(+)RNA	Flaviviridae	Japanese encephalitis virus	10976	3.03E-04	74	[18]
ss(+)RNA	Flaviviridae	Japanese encephalitis virus	10976	6.03E-04	74	[18]
ss(+)RNA	Flaviviridae	Kyasanur forest disease virus	10685	6.40E-04	49	[19]
ss(+)RNA	Flaviviridae	St. Louis encephalitis virus	10940	2.17E-04	78	[20]
ss(+)RNA	Flaviviridae	St. Louis encephalitis virus	10940	4.10E-04	72	[21]
ss(+)RNA	Flaviviridae	West Nile virus	11029	8.50E-04	8	[22]
ss(+)RNA	Luteoviridae	Barley yellow dwarf virus	5700	6.70E-03	89	[23]

ss(+)RNA	Luteoviridae	Barley yellow dwarf virus	5700	1.50E-03	89	[23]
ss(+)RNA	Luteoviridae	Barley yellow dwarf virus	5700	6.00E-04	89	[23]
ss(+)RNA	Luteoviridae	Barley yellow dwarf virus	5700	3.16E-04	2	[24]
ss(+)RNA	Luteoviridae	Beet chlorosis virus	5776	7.20E-03	20	[23]
ss(+)RNA	Luteoviridae	Beet chlorosis virus	5776	1.60E-03	20	[23]
ss(+)RNA	Luteoviridae	Beet chlorosis virus	5776	2.70E-03	20	[23]
ss(+)RNA	Luteoviridae	Beet mild yellowing virus	5722	7.70E-04	25	[23]
ss(+)RNA	Luteoviridae	Beet mild yellowing virus	5722	3.70E-04	25	[23]
ss(+)RNA	Luteoviridae	Beet mild yellowing virus	5722	4.90E-05	25	[23]
ss(+)RNA	Luteoviridae	Cereal yellow dwarf-RPV virus	5723	2.30E-02	81	[23]
ss(+)RNA	Luteoviridae	Cereal yellow dwarf-RPV virus	5723	7.40E-04	81	[23]
ss(+)RNA	Luteoviridae	Cereal yellow dwarf-RPV virus	5723	3.30E-03	81	[23]
ss(+)RNA	Luteoviridae	Cucurbit aphid-borne yellows virus	5669	4.30E-03	5	[23]
ss(+)RNA	Luteoviridae	Cucurbit aphid-borne yellows virus	5669	3.50E-02	5	[23]
ss(+)RNA	Luteoviridae	Potato leafroll virus	5987	3.40E-04	34	[23]
ss(+)RNA	Luteoviridae	Potato leafroll virus	5987	5.90E-05	34	[23]
ss(+)RNA	Luteoviridae	Potato leafroll virus	5987	6.20E-04	34	[23]
ss(+)RNA	Luteoviridae	Potato leafroll virus	5987	2.90E-04	34	[23]
ss(+)RNA	Luteoviridae	Soybean dwarf virus	5853	2.60E-03	17	[23]
ss(+)RNA	Luteoviridae	Soybean dwarf virus	5853	6.00E-04	17	[23]
ss(+)RNA	Luteoviridae	Soybean dwarf virus	5853	2.30E-03	17	[23]
ss(+)RNA	Luteoviridae	Sugarcane yellow leaf virus	5899	1.20E-03	19	[23]
ss(+)RNA	Luteoviridae	Sugarcane yellow leaf virus	5899	1.40E-04	19	[23]
ss(+)RNA	Luteoviridae	Sugarcane yellow leaf virus	5899	1.10E-03	19	[23]
ss(+)RNA	Luteoviridae	Sugarcane yellow leaf virus	5899	3.70E-05	19	[23]
ss(+)RNA	Luteoviridae	Turnip yellows virus	5641	2.70E-03	26	[23]
ss(+)RNA	Luteoviridae	Turnip yellows virus	5641	1.00E-03	26	[23]
ss(+)RNA	Luteoviridae	Turnip yellows virus	5641	8.60E-04	26	[23]
ss(+)RNA	Picornaviridae	Encephalomyocarditis virus	7835	1.61E-03	22	[25]
ss(+)RNA	Picornaviridae	Foot-and-mouth disease virus	8170	1.45E-03	38	[25]
ss(+)RNA	Picornaviridae	Foot-and-mouth disease virus	8170	1.46E-03	76	[26]
ss(+)RNA	Picornaviridae	Hepatitis A virus	7478	9.76E-04	17	[27]
ss(+)RNA	Picornaviridae	Hepatitis A virus	7478	8.90E-04	32	[25]
ss(+)RNA	Picornaviridae	Hepatitis A virus	7478	1.73E-04	33	[28]
ss(+)RNA	Picornaviridae	Human enterovirus A	7400	5.53E-03	24	[25]
ss(+)RNA	Picornaviridae	Human enterovirus A	7400	8.65E-03	59	[29]
ss(+)RNA	Picornaviridae	Human enterovirus A	7400	3.66E-03	29	[30]
ss(+)RNA	Picornaviridae	Human enterovirus A	7400	3.19E-03	62	[30]
ss(+)RNA	Picornaviridae	Human enterovirus B	7389	5.27E-03	62	[25]
ss(+)RNA	Picornaviridae	Human enterovirus B	7389	5.73E-03	48	[25]
ss(+)RNA	Picornaviridae	Human enterovirus B	7389	8.80E-03	49	[31]
ss(+)RNA	Picornaviridae	Human enterovirus B	7389	4.20E-03	26	[32]
ss(+)RNA	Picornaviridae	Human parechovirus	7348	2.96E-03	34	[25]
ss(+)RNA	Picornaviridae	Human parechovirus	7348	2.21E-03	51	[33]
ss(+)RNA	Picornaviridae	Human parechovirus	7348	2.79E-03	32	[33]
ss(+)RNA	Picornaviridae	Poliovirus 1	7440	1.17E-02	24	[25]

ss(+)RNA	Picornaviridae	Poliovirus 2	7440	1.01E-02	10	[34]
ss(+)RNA	Picornaviridae	Porcine teschovirus	7117	1.62E-03	50	[25]
ss(+)RNA	Potyviridae	Zucchini yellow mosaic virus	9591	5.00E-04	22	[35]
ss(+)RNA	Togaviridae	Fort Morgan virus	11381	2.64E-04	32	[36]
ss(+)RNA	Togaviridae	Fort Morgan virus	11381	1.81E-04	9	[36]
ss(+)RNA	Togaviridae	Ross River virus	11657	7.53E-04 (8.13E-04, 8.55E-04, 6.91E-04, 7.45E-04, 6.78E-04)	45	[37]
ss(+)RNA	Togaviridae	Venezuelan equine encephalitis virus	11444	1.28E-04	54	[38]
ss(+)RNA	Virgaviridae	Cucumber green mottle mosaic virus	6424	5.40E-05	38	[39]
ss(+)RNA	Virgaviridae	Cucumber green mottle mosaic virus	6424	6.70E-04	38	[39]
ss(+)RNA	Virgaviridae	Cucumber green mottle mosaic virus	6424	8.00E-04	38	[39]
ss(+)RNA	Virgaviridae	Odontoglossum ringspot virus	6618	7.70E-04	44	[39]
ss(+)RNA	Virgaviridae	Odontoglossum ringspot virus	6618	1.30E-03	44	[39]
ss(+)RNA	Virgaviridae	Odontoglossum ringspot virus	6618	7.80E-04	44	[39]
ss(+)RNA	Virgaviridae	Pepper mild mottle virus	6357	2.60E-04	36	[39]
ss(+)RNA	Virgaviridae	Pepper mild mottle virus	6357	8.90E-04	36	[39]
ss(+)RNA	Virgaviridae	Pepper mild mottle virus	6357	1.80E-04	36	[39]
ss(+)RNA	Virgaviridae	Ribgrass mosaic virus	6301	1.40E-04	51	[39]
ss(+)RNA	Virgaviridae	Tobacco mild green mosaic virus	6355	1.30E-04	101	[39]
ss(+)RNA	Virgaviridae	Tobacco mild green mosaic virus	6355	1.70E-04	101	[39]
ss(+)RNA	Virgaviridae	Tobacco mosaic virus	6395	2.90E-04	109	[39]
ss(+)RNA	Virgaviridae	Tobacco mosaic virus	6395	2.90E-04	109	[39]
ss(+)RNA	Virgaviridae	Tobacco mosaic virus	6395	1.60E-04	109	[39]
ss(+)RNA	Virgaviridae	Tomato mosaic virus	6383	2.10E-04	38	[39]
ss(+)RNA	none	Rice yellow mottle virus	4450	7.50E-04 (7.70E-04, 7.30E-04)	40	[40]
ss(-)RNA	Bunyaviridae	Crimean-Congo hemorrhagic fever virus	19146	1.09E-04	49	[41]
ss(-)RNA	Bunyaviridae	Crimean-Congo hemorrhagic fever virus	19146	1.52E-04	47	[41]
ss(-)RNA	Bunyaviridae	Crimean-Congo hemorrhagic fever virus	19146	5.80E-05	47	[41]
ss(-)RNA	Bunyaviridae	Hantaviruses virus	12305	6.76E-04	23	[42]
ss(-)RNA	Bunyaviridae	Rift valley fever virus	11979	2.35E-04	56	[43]
ss(-)RNA	Bunyaviridae	Rift valley fever virus	11979	2.42E-04	56	[43]
ss(-)RNA	Bunyaviridae	Rift valley fever virus	11979	2.78E-04	56	[43]
ss(-)RNA	Bunyaviridae	Sin Nombre virus	12317	6.76E-03	12	[44]
ss(-)RNA	Bunyaviridae	Sin Nombre virus	12317	1.93E-03	12	[44]
ss(-)RNA	Orthomyxoviridae	Influenza A virus	13588	3.15E-03	49	[45]
ss(-)RNA	Orthomyxoviridae	Influenza A virus	13588	2.86E-03	47	[45]
ss(-)RNA	Orthomyxoviridae	Influenza A virus	13588	3.48E-03	47	[45]
ss(-)RNA	Orthomyxoviridae	Influenza A virus	13588	3.92E-03	70	[45]
ss(-)RNA	Orthomyxoviridae	Influenza A virus	13588	3.17E-03	49	[45]
ss(-)RNA	Orthomyxoviridae	Influenza A virus	13588	3.61E-03	46	[45]

ss(-)RNA	Orthomyxoviridae	Influenza A virus	13588	2.49E-03	49	[45]
ss(-)RNA	Orthomyxoviridae	Influenza A virus	13588	3.87E-03	47	[45]
ss(-)RNA	Orthomyxoviridae	Influenza A virus	13588	3.28E-03	74	[46]
ss(-)RNA	Orthomyxoviridae	Influenza A virus	13588	1.82E-03	23	[46]
ss(-)RNA	Orthomyxoviridae	Influenza A virus	13588	2.11E-03	39	[46]
ss(-)RNA	Orthomyxoviridae	Influenza A virus	13588	3.12E-03	29	[46]
ss(-)RNA	Orthomyxoviridae	Influenza A virus	13588	3.20E-03	72	[46]
ss(-)RNA	Orthomyxoviridae	Influenza A virus	13588	4.31E-03	11	[46]
ss(-)RNA	Orthomyxoviridae	Influenza A virus	13588	7.84E-03	1	[47]
ss(-)RNA	Orthomyxoviridae	Influenza A virus	13588	1.98E-03	91	[48]
ss(-)RNA	Orthomyxoviridae	Influenza A virus	13588	3.43E-03	2	[48]
ss(-)RNA	Orthomyxoviridae	Influenza A virus	13588	2.29E-03	79	[48]
ss(-)RNA	Orthomyxoviridae	Influenza A virus	13588	3.41E-03	82	[48]
ss(-)RNA	Orthomyxoviridae	Influenza A virus	13588	2.80E-03	30	[48]
ss(-)RNA	Orthomyxoviridae	Influenza A virus	13588	2.25E-03	55	[48]
ss(-)RNA	Orthomyxoviridae	Influenza A virus	13588	9.00E-04	45	[48]
ss(-)RNA	Paramyxoviridae	Avian metapneumovirus	14071	4.47E-03	9	[49]
ss(-)RNA	Paramyxoviridae	Avian metapneumovirus	14071	7.01E-03	9	[49]
ss(-)RNA	Paramyxoviridae	Avian metapneumovirus	14071	1.39E-03	9	[49]
ss(-)RNA	Paramyxoviridae	Avian metapneumovirus	14071	6.14E-03	9	[49]
ss(-)RNA	Paramyxoviridae	Canine distemper virus	15690	1.17E-03	19	[50]
ss(-)RNA	Paramyxoviridae	Human parapneumovirus	13335	7.12E-04	25	[51]
ss(-)RNA	Paramyxoviridae	Measles virus	15894	6.02E-04	55	[52]
ss(-)RNA	Paramyxoviridae	Measles virus	15894	6.44E-04	55	[52]
ss(-)RNA	Paramyxoviridae	Measles virus	15894	6.58E-04	29	[50]
ss(-)RNA	Paramyxoviridae	Measles virus	15894	8.69E-04	26	[50]
ss(-)RNA	Paramyxoviridae	Mumps virus	15384	9.17E-04	54	[50]
ss(-)RNA	Paramyxoviridae	Respiratory syncytial virus	15191	2.31E-03	10	[53]
ss(-)RNA	Rhabdoviridae	European bat lyssavirus	12000	5.10E-05	35	[54]
ss(-)RNA	Rhabdoviridae	European bat lyssavirus	12000	6.11E-05	35	[54]
ss(-)RNA	Rhabdoviridae	Rabies virus	11932	3.32E-04	30	[55]
ss(-)RNA	Rhabdoviridae	Rabies virus	11932	2.57E-04	30	[55]
ss(-)RNA	Rhabdoviridae	Rabies virus	11932	3.57E-04	30	[55]
ss(-)RNA	Rhabdoviridae	Rabies virus	11932	2.75E-04	30	[55]
ss(-)RNA	Rhabdoviridae	Rabies virus	11932	3.90E-04	30	[55]
ss(-)RNA	Rhabdoviridae	Rabies virus	11932	2.59E-04	30	[55]
ss(-)RNA	Rhabdoviridae	Rabies virus	11932	1.38E-03	14	[56]
ss(-)RNA	Rhabdoviridae	Rabies virus	11932	2.32E-04	8	[56]
ss(-)RNA	Rhabdoviridae	Rabies virus	11932	2.70E-04	33	[57]
ss(-)RNA	Rhabdoviridae	Rabies virus	11932	1.68E-03	13	[58]
ss(-)RNA	Rhabdoviridae	Rabies virus	11932	8.26E-04	20	[58]
ss(-)RNA	Rhabdoviridae	Rabies virus	11932	1.53E-04	40	[59]
ss(-)RNA	Rhabdoviridae	Rabies virus	11932	3.96E-04	39	[60]
dsRNA	Birnaviridae	Infectious bursal disease virus	5898	6.36E-04 (6.40E-04, 6.00E-04, 6.70E-04)	12	[61]
dsRNA	Reoviridae	Bluetongue virus	19186	3.71E-04 (2.79E-04, 4.94E-04)	45	[62]
dsRNA	Reoviridae	Bluetongue virus	19186	1.79E-04 (1.68E-04, 1.90E-04)	44	[62]
dsRNA	Reoviridae	Bluetongue virus	19186	5.37E-05 (5.52E-05, 5.22E-05)	37	[62]
dsRNA	Reoviridae	Bluetongue virus	19186	5.39E-04	28	[62]

				(4.19E-04, 6.94E-04)		
dsRNA	Reoviridae	Homalodisca vitripennis virus	25724	3.46E-05 (5.42E-05, 3.30E-05, 2.32E-05)	6	[63]
dsRNA	Reoviridae	Human rotavirus A	18555	1.87E-03	29	[64]
dsRNA	Reoviridae	Human rotavirus A	18555	1.66E-03	21	[64]
dsRNA	Reoviridae	Liao ning virus	20739	1.99E-03	2	[65]
RT	Hepadnaviridae	Avian hepatitis B virus	3027	7.32E-04	16	[66]
RT	Hepadnaviridae	Human hepatitis B virus	3215	7.19E-04 (6.01E-04, 8.60E-04)	30	[67]
RT	Hepadnaviridae	Human hepatitis B virus	3215	1.57E-04 (1.23E-04, 2.00E-04)	30	[67]
RT	Hepadnaviridae	Human hepatitis B virus	3215	1.11E-04 (1.01E-04, 1.21E-04)	30	[67]
RT	Hepadnaviridae	Human hepatitis B virus	3215	4.25E-04 (1.94E-04, 9.29E-04)	30	[67]
RT	Hepadnaviridae	Human hepatitis B virus	3215	7.66E-04 (5.29E-04, 1.11E-03)	30	[67]
RT	Hepadnaviridae	Human hepatitis B virus	3215	1.12E-04 (4.39E-05, 2.88E-04)	30	[67]
RT	Hepadnaviridae	Human hepatitis B virus	3215	7.72E-04	23	[66]
RT	Retroviridae	HIV-1	9749	2.50E-03	11	[68]
RT	Retroviridae	HIV-1	9749	1.80E-03	12	[68]
RT	Retroviridae	HIV-1	9749	2.06E-03	15	[69]
RT	Retroviridae	HIV-1	9749	1.89E-03	2	[70]
RT	Retroviridae	HIV-1	9749	1.55E-03	2	[70]
RT	Retroviridae	HIV-1	9749	1.42E-03	2	[70]
RT	Retroviridae	HIV-1	9749	1.55E-03	2	[70]
RT	Retroviridae	HIV-1	9749	1.13E-03	2	[70]
RT	Retroviridae	HIV-1	9749	2.41E-03	2	[70]
RT	Retroviridae	HIV-1	9749	2.56E-03	2	[70]
RT	Retroviridae	HIV-1	9749	3.63E-03	2	[70]
RT	Retroviridae	HIV-1	9749	2.46E-03	2	[70]
RT	Retroviridae	HIV-1	9749	2.55E-03	2	[70]
RT	Retroviridae	HIV-1	9749	1.08E-02	7	[71]
ssDNA	Circoviridae	Porcine circovirus 2	1768	1.15E-03	9	[72]
ssDNA	Circoviridae	Porcine circovirus 2	1768	1.64E-03	25	[72]
ssDNA	Geminiviridae	African cassava mosaic virus	5503	1.60E-03	7	[73]
ssDNA	Geminiviridae	African cassava mosaic virus	5503	1.33E-04	7	[73]
ssDNA	Geminiviridae	African cassava mosaic virus	5503	1.56E-03	7	[74]
ssDNA	Geminiviridae	Maize streak virus	2690	3.87E-04	29	[74]
ssDNA	Geminiviridae	Tomato yellow leaf curl virus	2781	2.88E-04	18	[75]
ssDNA	Geminiviridae	Tomato yellow leaf curl virus	2781	4.04E-04	18	[74]
ssDNA	Nanoviridae	Banana bunchy top virus	6396	1.43E-04	16	[76]
ssDNA	Parvoviridae	Canine parvovirus	5323	1.70E-04	26	[77]
ssDNA	Parvoviridae	Canine parvovirus	5323	7.90E-05	26	[77]
ssDNA	Parvoviridae	Feline panleukopenia virus	5323	9.40E-05	34	[77]
ssDNA	Parvoviridae	Feline panleukopenia virus	5323	7.90E-05	28	[77]
ssDNA	Parvoviridae	Human parvovirus B19	5594	1.14E-04	28	[78]
ssDNA	Parvoviridae	Human parvovirus B19	5594	1.83E-04	28	[78]

ssDNA	Parvoviridae	Human parvovirus B19	5594	1.20E-04	14	[79]
ssDNA	Parvoviridae	Human parvovirus B19	5594	2.30E-04	14	[79]
ssDNA	Parvoviridae	Infectious hypodermal and hematopoietic necrosis virus	3909	1.39E-04	21	[80]
ssDNA	Parvoviridae	Porcine parvovirus	5075	3.02E-04	33	[81]
ssDNA	Parvoviridae	Porcine parvovirus	5075	4.04E-04	33	[81]
ssDNA	Parvoviridae	Porcine parvovirus	5075	5.39E-05	33	[81]
dsDNA	Adenoviridae	Human adenovirus B	35343	7.20E-05	24	[82]
dsDNA	Adenoviridae	Human adenovirus C	35937	3.46E-05	21	[82]
dsDNA	Herpesviridae	Herpes simplex virus 1	152261	8.21E-05	27	[82]
dsDNA	Herpesviridae	Varicella zoster virus	124884	6.26E-06	37	[82]
dsDNA	Papillomaviridae	Human papillomavirus16	7905	3.94E-03	3	[82]
dsDNA	Polyomaviridae	BK polyomavirus	5153	4.34E-05	29	[82]
dsDNA	Polyomaviridae	JC polyomavirus	5130	1.70E-05	33	[83]
dsDNA	Poxviridae	Monkeypox virus	196858	7.00E-07	26	[84]
dsDNA	Poxviridae	Variola virus	185578	9.32E-06	31	[82]

*Genome size

†Values directly taken from the original study. When several estimates obtained by different methods were available for the same dataset and no best-fit value was provided, the geometric mean was used and original values are indicated in parentheses.

Table S2: Fitness effects of point mutations determined by site-directed mutagenesis

Virus	Site	q^*	Deleterious	Lethal	$B^\dagger$ (upper)	$s^\ddagger$ (upper)	B (lower)	s (lower)	Ref.
Bacteriophage Qβ	161	0.993	1	0	4000	0.054	400	0.039	[85]
Bacteriophage Qβ	328	1.003	0	0	4000		400		[85]
Bacteriophage Qβ	665	0.560	1	0	4000	0.974	400	0.931	[85]
Bacteriophage Qβ	733	0.000	1	1	4000	1.000	400	1.000	[85]
Bacteriophage Qβ	772	0.635	1	0	4000	0.952	400	0.890	[85]
Bacteriophage Qβ	891	0.905	1	0	4000	0.545	400	0.435	[85]
Bacteriophage Qβ	939	0.928	1	0	4000	0.450	400	0.351	[85]
Bacteriophage Qβ	1096	0.974	1	0	4000	0.191	400	0.142	[85]
Bacteriophage Qβ	1149	0.000	1	1	4000	1.000	400	1.000	[85]
Bacteriophage Qβ	1165	0.961	1	0	4000	0.279	400	0.211	[85]
Bacteriophage Qβ	1283	0.000	1	1	4000	1.000	400	1.000	[85]
Bacteriophage Qβ	1288	0.000	1	1	4000	1.000	400	1.000	[85]
Bacteriophage Qβ	1329	0.000	1	1	4000	1.000	400	1.000	[85]
Bacteriophage Qβ	1442	0.000	1	1	4000	1.000	400	1.000	[85]
Bacteriophage Qβ	1485	0.957	1	0	4000	0.303	400	0.230	[85]
Bacteriophage Qβ	1525	1.035	0	0	4000		400		[85]
Bacteriophage Qβ	1809	0.973	1	0	4000	0.201	400	0.150	[85]
Bacteriophage Qβ	1814	0.986	1	0	4000	0.108	400	0.080	[85]
Bacteriophage Qβ	1895	0.941	1	0	4000	0.385	400	0.297	[85]
Bacteriophage Qβ	2090	0.770	1	0	4000	0.852	400	0.750	[85]
Bacteriophage Qβ	2360	0.000	1	1	4000	1.000	400	1.000	[85]
Bacteriophage Qβ	2379	0.000	1	1	4000	1.000	400	1.000	[85]
Bacteriophage Qβ	2787	0.902	1	0	4000	0.558	400	0.446	[85]
Bacteriophage Qβ	2793	0.907	1	0	4000	0.539	400	0.430	[85]
Bacteriophage Qβ	2797	0.919	1	0	4000	0.491	400	0.387	[85]
Bacteriophage Qβ	3027	1.025	0	0	4000		400		[85]
Bacteriophage Qβ	3075	1.000	0	0	4000		400		[85]
Bacteriophage Qβ	3094	1.007	0	0	4000		400		[85]
Bacteriophage Qβ	3101	0.000	1	1	4000	1.000	400	1.000	[85]
Bacteriophage Qβ	3210	0.000	1	1	4000	1.000	400	1.000	[85]
Bacteriophage Qβ	3213	0.953	1	0	4000	0.325	400	0.248	[85]
Bacteriophage Qβ	3397	0.740	1	0	4000	0.884	400	0.791	[85]
Bacteriophage Qβ	3415	0.000	1	1	4000	1.000	400	1.000	[85]
Bacteriophage Qβ	3452	0.671	1	0	4000	0.935	400	0.863	[85]
Bacteriophage Qβ	3483	1.016	0	0	4000		400		[85]
Bacteriophage Qβ	3661	0.777	1	0	4000	0.843	400	0.739	[85]
Bacteriophage Qβ	3686	0.000	1	1	4000	1.000	400	1.000	[85]
Bacteriophage Qβ	3815	1.032	0	0	4000		400		[85]
Bacteriophage Qβ	3916	0.772	1	0	4000	0.849	400	0.747	[85]
Bacteriophage Qβ	3951	0.996	1	0	4000	0.034	400	0.025	[85]
Bacteriophage Qβ	4066	0.931	1	0	4000	0.437	400	0.340	[85]
Bacteriophage Qβ	4082	0.648	1	0	4000	0.946	400	0.881	[85]
Vesicular stomatitis virus	112	0.000	1	1	1000	1.000	100	1.000	[86]
Vesicular stomatitis virus	636	0.000	1	1	1000	1.000	100	1.000	[86]
Vesicular stomatitis virus	877	0.831	1	0	1000	0.690	100	0.546	[86]
Vesicular stomatitis virus	906	1.055	0	0	1000		100		[86]

Vesicular stomatitis virus	1044	1.002	0	0	1000		100		[86]
Vesicular stomatitis virus	1073	0.265	1	0	1000	0.995	100	0.976	[86]
Vesicular stomatitis virus	1323	0.429	1	0	1000	0.982	100	0.937	[86]
Vesicular stomatitis virus	1585	0.965	1	0	1000	0.214	100	0.150	[86]
Vesicular stomatitis virus	2018	0.961	1	0	1000	0.239	100	0.168	[86]
Vesicular stomatitis virus	2613	1.002	0	0	1000		100		[86]
Vesicular stomatitis virus	2767	0.000	1	1	1000	1.000	100	1.000	[86]
Vesicular stomatitis virus	2877	0.000	1	1	1000	1.000	100	1.000	[86]
Vesicular stomatitis virus	2969	0.988	1	0	1000	0.082	100	0.056	[86]
Vesicular stomatitis virus	2969	0.954	1	0	1000	0.275	100	0.195	[86]
Vesicular stomatitis virus	3079	0.000	1	1	1000	1.000	100	1.000	[86]
Vesicular stomatitis virus	3192	0.571	1	0	1000	0.949	100	0.870	[86]
Vesicular stomatitis virus	3327	0.000	1	1	1000	1.000	100	1.000	[86]
Vesicular stomatitis virus	3446	0.961	1	0	1000	0.237	100	0.167	[86]
Vesicular stomatitis virus	3615	0.877	1	0	1000	0.574	100	0.438	[86]
Vesicular stomatitis virus	3625	0.000	1	1	1000	1.000	100	1.000	[86]
Vesicular stomatitis virus	3688	0.000	1	1	1000	1.000	100	1.000	[86]
Vesicular stomatitis virus	3950	0.916	1	0	1000	0.440	100	0.323	[86]
Vesicular stomatitis virus	4163	0.000	1	1	1000	1.000	100	1.000	[86]
Vesicular stomatitis virus	4175	1.020	0	0	1000		100		[86]
Vesicular stomatitis virus	4398	0.875	1	0	1000	0.580	100	0.443	[86]
Vesicular stomatitis virus	4459	0.732	1	0	1000	0.844	100	0.716	[86]
Vesicular stomatitis virus	4824	0.000	1	1	1000	1.000	100	1.000	[86]
Vesicular stomatitis virus	4984	1.018	0	0	1000		100		[86]
Vesicular stomatitis virus	5124	0.774	1	0	1000	0.790	100	0.653	[86]
Vesicular stomatitis virus	5496	1.005	0	0	1000		100		[86]
Vesicular stomatitis virus	5597	1.030	0	0	1000		100		[86]
Vesicular stomatitis virus	6010	0.947	1	0	1000	0.307	100	0.219	[86]
Vesicular stomatitis virus	6676	0.000	1	1	1000	1.000	100	1.000	[86]
Vesicular stomatitis virus	6907	0.000	1	1	1000	1.000	100	1.000	[86]
Vesicular stomatitis virus	7123	0.947	1	0	1000	0.305	100	0.217	[86]
Vesicular stomatitis virus	7128	0.635	1	0	1000	0.920	100	0.822	[86]
Vesicular stomatitis virus	7287	0.681	1	0	1000	0.890	100	0.778	[86]
Vesicular stomatitis virus	7739	0.956	1	0	1000	0.262	100	0.185	[86]
Vesicular stomatitis virus	7918	0.967	1	0	1000	0.204	100	0.142	[86]
Vesicular stomatitis virus	8188	0.000	1	1	1000	1.000	100	1.000	[86]
Vesicular stomatitis virus	8374	0.000	1	1	1000	1.000	100	1.000	[86]
Vesicular stomatitis virus	8426	0.000	1	1	1000	1.000	100	1.000	[86]
Vesicular stomatitis virus	8459	0.880	1	0	1000	0.565	100	0.430	[86]
Vesicular stomatitis virus	8787	0.937	1	0	1000	0.351	100	0.253	[86]
Vesicular stomatitis virus	9315	0.000	1	1	1000	1.000	100	1.000	[86]
Vesicular stomatitis virus	9535	0.000	1	1	1000	1.000	100	1.000	[86]
Vesicular stomatitis virus	10595	0.000	1	1	1000	1.000	100	1.000	[86]
Vesicular stomatitis virus	10941	0.000	1	1	1000	1.000	100	1.000	[86]
Tobacco etch virus	158	0.916	1	0	6	0.167	3	0.132	[87]
Tobacco etch virus	337	0.587	1	0	6	0.627	3	0.547	[87]
Tobacco etch virus	375	0.659	1	0	6	0.548	3	0.468	[87]
Tobacco etch virus	475	0.928	1	0	6	0.146	3	0.114	[87]
Tobacco etch virus	872	0.972	1	0	6	0.059	3	0.045	[87]
Tobacco etch virus	1503	1.346	0	0	6		3		[87]

Tobacco etch virus	1569	0.585	1	0	6	0.630	3	0.549	[87]
Tobacco etch virus	1655	0.742	1	0	6	0.444	3	0.370	[87]
Tobacco etch virus	1758	0.000	1	1	6	1.000	3	1.000	[87]
Tobacco etch virus	1851	0.828	1	0	6	0.319	3	0.259	[87]
Tobacco etch virus	2119	0.759	1	0	6	0.420	3	0.348	[87]
Tobacco etch virus	2171	0.000	1	1	6	1.000	3	1.000	[87]
Tobacco etch virus	2216	0.000	1	1	6	1.000	3	1.000	[87]
Tobacco etch virus	2317	0.000	1	1	6	1.000	3	1.000	[87]
Tobacco etch virus	2412	0.000	1	1	6	1.000	3	1.000	[87]
Tobacco etch virus	2572	0.000	1	1	6	1.000	3	1.000	[87]
Tobacco etch virus	2750	0.000	1	1	6	1.000	3	1.000	[87]
Tobacco etch virus	2967	0.000	1	1	6	1.000	3	1.000	[87]
Tobacco etch virus	2977	1.264	0	0	6		3		[87]
Tobacco etch virus	3154	0.000	1	1	6	1.000	3	1.000	[87]
Tobacco etch virus	3179	0.000	1	1	6	1.000	3	1.000	[87]
Tobacco etch virus	3238	0.905	1	0	6	0.188	3	0.149	[87]
Tobacco etch virus	3406	0.973	1	0	6	0.057	3	0.044	[87]
Tobacco etch virus	3462	0.000	1	1	6	1.000	3	1.000	[87]
Tobacco etch virus	3468	0.800	1	0	6	0.362	3	0.297	[87]
Tobacco etch virus	3900	1.114	0	0	6		3		[87]
Tobacco etch virus	4101	1.235	0	0	6		3		[87]
Tobacco etch virus	4418	0.931	1	0	6	0.139	3	0.109	[87]
Tobacco etch virus	4510	0.000	1	1	6	1.000	3	1.000	[87]
Tobacco etch virus	4570	0.715	1	0	6	0.479	3	0.403	[87]
Tobacco etch virus	4828	0.000	1	1	6	1.000	3	1.000	[87]
Tobacco etch virus	5038	0.000	1	1	6	1.000	3	1.000	[87]
Tobacco etch virus	5115	0.561	1	0	6	0.653	3	0.574	[87]
Tobacco etch virus	5254	0.000	1	1	6	1.000	3	1.000	[87]
Tobacco etch virus	5279	0.000	1	1	6	1.000	3	1.000	[87]
Tobacco etch virus	5336	0.000	1	1	6	1.000	3	1.000	[87]
Tobacco etch virus	5344	0.900	1	0	6	0.198	3	0.157	[87]
Tobacco etch virus	5349	0.970	1	0	6	0.063	3	0.048	[87]
Tobacco etch virus	5351	0.803	1	0	6	0.357	3	0.292	[87]
Tobacco etch virus	5387	0.872	1	0	6	0.246	3	0.196	[87]
Tobacco etch virus	5582	0.964	1	0	6	0.074	3	0.058	[87]
Tobacco etch virus	5793	0.610	1	0	6	0.603	3	0.523	[87]
Tobacco etch virus	5908	0.000	1	1	6	1.000	3	1.000	[87]
Tobacco etch virus	5924	0.000	1	1	6	1.000	3	1.000	[87]
Tobacco etch virus	6012	0.924	1	0	6	0.152	3	0.120	[87]
Tobacco etch virus	6044	0.963	1	0	6	0.077	3	0.060	[87]
Tobacco etch virus	6197	1.229	0	0	6		3		[87]
Tobacco etch virus	6251	0.708	1	0	6	0.488	3	0.411	[87]
Tobacco etch virus	6409	0.942	1	0	6	0.119	3	0.093	[87]
Tobacco etch virus	6418	0.000	1	1	6	1.000	3	1.000	[87]
Tobacco etch virus	6519	1.025	0	0	6		3		[87]
Tobacco etch virus	6660	0.930	1	0	6	0.141	3	0.111	[87]
Tobacco etch virus	6890	0.000	1	1	6	1.000	3	1.000	[87]
Tobacco etch virus	6988	0.753	1	0	6	0.429	3	0.356	[87]
Tobacco etch virus	7034	0.000	1	1	6	1.000	3	1.000	[87]
Tobacco etch virus	7182	1.158	0	0	6		3		[87]

Tobacco etch virus	7315	0.918	1	0	6	0.163	3	0.129	[87]
Tobacco etch virus	7582	0.000	1	1	6	1.000	3	1.000	[87]
Tobacco etch virus	7643	0.000	1	1	6	1.000	3	1.000	[87]
Tobacco etch virus	8026	0.000	1	1	6	1.000	3	1.000	[87]
Tobacco etch virus	8359	1.141	0	0	6		3		[87]
Tobacco etch virus	8398	0.812	1	0	6	0.344	3	0.280	[87]
Tobacco etch virus	8501	0.000	1	1	6	1.000	3	1.000	[87]
Tobacco etch virus	8988	0.589	1	0	6	0.625	3	0.545	[87]
Tobacco etch virus	9256	0.605	1	0	6	0.608	3	0.528	[87]
Tobacco etch virus	9466	0.000	1	1	6	1.000	3	1.000	[87]

[*] For bacteriophage Qβ and vesicular stomatitis virus, fitness was determined as the mutant to wild-type growth rate ratio, *q*. For tobacco etch virus, a different unit was originally used, but data were converted to growth rate ratios as detailed in previous work (88).

[†] Per-cell burst sizes. Since these are uncertain, lower- and upper-limits based were used (88).

[‡] Selection coefficient per cell infection, calculated as $s = 1-(B^q-1)/(B-1)$. *s* (lower) and *s* (upper) refer to *s* calculated using lower and upper limit *B* values, respectively. *s*>0 means that the mutation is deleterious; *s* = 1 means that the mutation is lethal; *s* is not shown for beneficial mutations. Values given in the maim text correspond to the harmonic mean of all deleterious selection coefficients, pooling the three datasets. This calculation was done including or excluding lethal mutations, as well as using upper- or lower-limit *B* values, hence defining the intervals indicated in the main text.

Supplementary References

1. Song J, Shen D, Cui J, Zhao B (2010) Accelerated evolution of PRRSV during recent outbreaks in China. Virus Genes 41: 241-245.
2. Bok K et al. (2009) Evolutionary dynamics of GII.4 noroviruses over a 34-year period. J Virol 83: 11890-11901.
3. Boon D et al. (2011) Comparative evolution of GII.3 and GII.4 norovirus over a 31-year period. J Virol 85: 8656-8666.
4. Kerr PJ, Kitchen A, Holmes EC (2009) Origin and phylodynamics of rabbit hemorrhagic disease virus. J Virol 83: 12129-12138.
5. McKinley ET et al. (2011) Attenuated live vaccine usage affects accurate measures of virus diversity and mutation rates in avian coronavirus infectious bronchitis virus. Virus Res 158: 225-234.
6. Vijgen L et al. (2005) Complete genomic sequence of human coronavirus OC43: molecular clock analysis suggests a relatively recent zoonotic coronavirus transmission event. J Virol 79: 1595-1604.
7. Pyrc K et al. (2006) Mosaic structure of human coronavirus NL63, one thousand years of evolution. J Mol Biol 364: 964-973.
8. Hon CC et al. (2008) Evidence of the recombinant origin of a bat severe acute respiratory syndrome (SARS)-like coronavirus and its implications on the direct ancestor of SARS coronavirus. J Virol 82: 1819-1826.
9. Lau SK et al. (2010) Ecoepidemiology and complete genome comparison of different strains of severe acute respiratory syndrome-related Rhinolophus bat coronavirus in China reveal bats as a reservoir for acute, self-limiting infection that allows recombination events. J Virol 84: 2808-2819.
10. Wertheim JO, Tang KF, Navarro SA, Lightner DV (2009) A quick fuse and the emergence of Taura syndrome virus. Virology 390: 324-329.
11. Patil JA et al. (2011) Evolutionary dynamics of the American African genotype of dengue type 1 virus in India (1962-2005). Infect Genet Evol 11: 1443-1448.

12. Klungthong C, Zhang C, Mammen MP, Jr., Ubol S, Holmes EC (2004) The molecular epidemiology of dengue virus serotype 4 in Bangkok, Thailand. Virology 329: 168-179.

13. Kumar SR et al. (2010) Evolution, dispersal and replacement of American genotype dengue type 2 viruses in India (1956-2005): selection pressure and molecular clock analyses. J Gen Virol 91: 707-720.

14. Ramirez A et al. (2010) Evolution of dengue virus type 3 genotype III in Venezuela: diversification, rates and population dynamics. Virol J 7: 329.

15. Romano CM, Zanotto PM, Holmes EC (2008) Bayesian coalescent analysis reveals a high rate of molecular evolution in GB virus C. J Mol Evol 66: 292-297.

16. Gray RR et al. (2011) The mode and tempo of hepatitis C virus evolution within and among hosts. BMC Evol Biol 11: 131-11.

17. Magiorkinis G et al. (2009) The global spread of hepatitis C virus 1a and 1b: a phylodynamic and phylogeographic analysis. PLoS Med 6: e1000198.

18. Mohammed MA et al. (2011) Molecular phylogenetic and evolutionary analyses of Muar strain of Japanese encephalitis virus reveal it is the missing fifth genotype. Infect Genet Evol 11: 855-862.

19. Mehla R et al. (2009) Recent ancestry of Kyasanur Forest disease virus. Emerg Infect Dis 15: 1431-1437.

20. Baillie GJ et al. (2008) Phylogenetic and evolutionary analyses of St. Louis encephalitis virus genomes. Mol Phylogenet Evol 47: 717-728.

21. Auguste AJ, Pybus OG, Carrington CV (2009) Evolution and dispersal of St. Louis encephalitis virus in the Americas. Infect Genet Evol 9: 709-715.

22. Bertolotti L, Kitron U, Goldberg TL (2007) Diversity and evolution of West Nile virus in Illinois and the United States, 2002-2005. Virology 360: 143-149.

23. Pagan I, Holmes EC (2010) Long-term evolution of the Luteoviridae: time scale and mode of virus speciation. J Virol 84: 6177-6187.

24. Wu B et al. (2011) Dynamics of molecular evolution and phylogeography of Barley yellow dwarf virus-PAV. PLoS ONE 6: e16896.

25. Hicks AL, Duffy S (2011) Genus-specific substitution rate variability among picornaviruses. J Virol 85: 7942-7947.

26. Yoon SH, Park W, King DP, Kim H (2011) Phylogenomics and molecular evolution of foot-and-mouth disease virus. Mol Cells 31: 413-421.

27. Moratorio G et al. (2007) Bayesian coalescent inference of hepatitis A virus populations: evolutionary rates and patterns. J Gen Virol 88: 3039-3042.

28. Kulkarni MA, Walimbe AM, Cherian S, Arankalle VA (2009) Full length genomes of genotype IIIA Hepatitis A Virus strains (1995-2008) from India and estimates of the evolutionary rates and ages. Infect Genet Evol 9: 1287-1294.

29. Chu PY et al. (2011) Spatiotemporal phylogenetic analysis and molecular characterization of coxsackievirus A4. Infect Genet Evol 11: 1426-1435.

30. Mirand A et al. (2010) Phylogenetic evidence for a recent spread of two populations of human enterovirus 71 in European countries. J Gen Virol 91: 2263-2277.

31. McWilliam Leitch EC et al. (2009) Transmission networks and population turnover of echovirus 30. J Virol 83: 2109-2118.

32. Gullberg M et al. (2010) Characterization of a putative ancestor of coxsackievirus B5. J Virol 84: 9695-9708.

33. Faria NR, de Vries M, van Hemert FJ, Benschop K, van der HL (2009) Rooting human parechovirus evolution in time. BMC Evol Biol 9: 164.

34. Jorba J, Campagnoli R, De L, Kew O (2008) Calibration of multiple poliovirus molecular clocks covering an extended evolutionary range. J Virol 82: 4429-4440.

35. Simmons HE, Holmes EC, Stephenson AG (2008) Rapid evolutionary dynamics of zucchini yellow mosaic virus. J Gen Virol 89: 1081-1085.

36. Padhi A et al. (2008) Phylogeographical structure and evolutionary history of two Buggy Creek virus lineages in the western Great Plains of North America. J Gen Virol 89: 2122-2131.

37. Jones A, Lowry K, Aaskov J, Holmes EC, Kitchen A (2010) Molecular evolutionary dynamics of Ross River virus and implications for vaccine efficacy. J Gen Virol 91: 182-188.

38. Auguste AJ et al. (2009) Isolation and phylogenetic analysis of Mucambo virus (Venezuelan equine encephalitis complex subtype IIIA) in Trinidad. Virology 392: 123-130.

39. Pagan I, Firth C, Holmes EC (2010) Phylogenetic analysis reveals rapid evolutionary dynamics in the plant RNA virus genus tobamovirus. J Mol Evol 71: 298-307.

40. Fargette D et al. (2008) Rice yellow mottle virus, an RNA plant virus, evolves as rapidly as most RNA animal viruses. J Virol 82: 3584-3589.

41. Carroll SA, Bird BH, Rollin PE, Nichol ST (2010) Ancient common ancestry of Crimean-Congo hemorrhagic fever virus. Mol Phylogenet Evol 55: 1103-1110.

42. Ramsden C, Holmes EC, Charleston MA (2009) Hantavirus evolution in relation to its rodent and insectivore hosts: no evidence for codivergence. Mol Biol Evol 26: 143-153.

43. Bird BH, Khristova ML, Rollin PE, Ksiazek TG, Nichol ST (2007) Complete genome analysis of 33 ecologically and biologically diverse Rift Valley fever virus strains reveals widespread virus movement and low genetic diversity due to recent common ancestry. J Virol 81: 2805-2816.

44. Black WC, Doty JB, Hughes MT, Beaty BJ, Calisher CH (2009) Temporal and geographic evidence for evolution of Sin Nombre virus using molecular analyses of viral RNA from Colorado, New Mexico and Montana. Virol J 6: 102.

45. Chen R, Holmes EC (2006) Avian influenza virus exhibits rapid evolutionary dynamics. Mol Biol Evol 23: 2336-2341.

46. Fourment M, Wood JT, Gibbs AJ, Gibbs MJ (2010) Evolutionary dynamics of the N1 neuraminidases of the main lineages of influenza A viruses. Mol Phylogenet Evol 56: 526-535.

47. Goni N, Fajardo A, Moratorio G, Colina R, Cristina J (2009) Modeling gene sequences over time in 2009 H1N1 influenza A virus populations. Virol J 6: 215.

48. Xu J, Christman MC, Donis RO, Lu G (2011) Evolutionary dynamics of influenza A nucleoprotein (NP) lineages revealed by large-scale sequence analyses. Infect Genet Evol, in press.

49. Padhi A, Poss M (2009) Population dynamics and rates of molecular evolution of a recently emerged paramyxovirus, avian metapneumovirus subtype C. J Virol 83: 2015-2019.

50. Pomeroy LW, Bjornstad ON, Holmes EC (2008) The evolutionary and epidemiological dynamics of the paramyxoviridae. J Mol Evol 66: 98-106.

51. Yang CF et al. (2009) Genetic diversity and evolution of human metapneumovirus fusion protein over twenty years. Virol J 6: 138.

52. Furuse Y, Suzuki A, Oshitani H (2010) Origin of measles virus: divergence from rinderpest virus between the 11th and 12th centuries. Virol J 7: 52.

53. van Niekerk S, Venter M (2011) Replacement of previously circulating respiratory syncytial virus subtype B strains with the BA genotype in South Africa. J Virol 85: 8789-8797.

54. Davis PL et al. (2005) Phylogeography, population dynamics, and molecular evolution of European bat lyssaviruses. J Virol 79: 10487-10497.

55. Davis PL, Bourhy H, Holmes EC (2006) The evolutionary history and dynamics of bat rabies virus. Infect Genet Evol 6: 464-473.

56. Hughes GJ, Orciari LA, Rupprecht CE (2005) Evolutionary timescale of rabies virus adaptation to North American bats inferred from the substitution rate of the nucleoprotein gene. J Gen Virol 86: 1467-1474.

57. David D et al. (2007) Identification of novel canine rabies virus clades in the Middle East and North Africa. J Gen Virol 88: 967-980.

58. Davis PL, Rambaut A, Bourhy H, Holmes EC (2007) The evolutionary dynamics of canid and mongoose rabies virus in Southern Africa. Arch Virol 152: 1251-1258.

59. Meng S et al. (2010) Transmission dynamics of rabies in China over the last 40 years: 1969-2009. J Clin Virol 49: 47-52.

60. Ming P et al. (2010) A history estimate and evolutionary analysis of rabies virus variants in China. J Gen Virol 91: 759-764.

61. Hon CC et al. (2006) Phylogenetic analysis reveals a correlation between the expansion of very virulent infectious bursal disease virus and reassortment of its genome segment B. J Virol 80: 8503-8509.

62. Carpi G, Holmes EC, Kitchen A (2010) The evolutionary dynamics of bluetongue virus. J Mol Evol 70: 583-592.

63. Stenger DC, Sisterson MS, French R (2010) Population genetics of Homalodisca vitripennis reovirus validates timing and limited introduction to California of its invasive insect host, the glassy-winged sharpshooter. Virology 407: 53-59.

64. Matthijnssens J et al. (2010) Phylodynamic analyses of rotavirus genotypes G9 and G12 underscore their potential for swift global spread. Mol Biol Evol 27: 2431-2436.

65. Lu Z et al. (2011) Liao ning virus in China. Virol J 8: 282.

66. Zhou Y, Holmes EC (2007) Bayesian estimates of the evolutionary rate and age of hepatitis B virus. J Mol Evol 65: 197-205.

67. Harrison A et al. (2011) Genomic analysis of hepatitis B virus reveals antigen state and genotype as sources of evolutionary rate variation. Viruses 3: 83-101.

68. Bello G et al. (2010) Phylodynamics of HIV-1 circulating recombinant forms 12_BF and 38_BF in Argentina and Uruguay. Retrovirology 7: 22.

69. Dalai SC et al. (2009) Evolution and molecular epidemiology of subtype C HIV-1 in Zimbabwe. AIDS 23: 2523-2532.

70. Lemey P, Van Dooren S, Vandamme AM (2005) Evolutionary dynamics of human retroviruses investigated through full-genome scanning. Mol Biol Evol 22: 942-951.

71. Aulicino PC, Holmes EC, Rocco C, Mangano A, Sen L (2007) Extremely rapid spread of human immunodeficiency virus type 1 BF recombinants in Argentina. J Virol 81: 427-429.

72. Firth C, Charleston MA, Duffy S, Shapiro B, Holmes EC (2009) Insights into the evolutionary history of an emerging livestock pathogen: porcine circovirus 2. J Virol 83: 12813-12821.

73. Duffy S, Holmes EC (2009) Validation of high rates of nucleotide substitution in geminiviruses: phylogenetic evidence from East African cassava mosaic viruses. J Gen Virol 90: 1539-1547.

74. Lefeuvre P et al. (2011) Evolutionary time-scale of the begomoviruses: evidence from integrated sequences in the Nicotiana genome. PLoS ONE 6: e19193.

75. Duffy S, Holmes EC (2008) Phylogenetic evidence for rapid rates of molecular evolution in the single-stranded DNA begomovirus tomato yellow leaf curl virus. J Virol 82: 957-965.

76. Almeida RP, Bennett GM, Anhalt MD, Tsai CW, O'Grady P (2009) Spread of an introduced vector-borne banana virus in Hawaii. Mol Ecol 18: 136-146.

77. Shackelton LA, Parrish CR, Truyen U, Holmes EC (2005) High rate of viral evolution associated with the emergence of carnivore parvovirus. Proc Natl Acad Sci U SA 102: 379-384.

78. Shackelton LA, Holmes EC (2006) Phylogenetic evidence for the rapid evolution of human B19 erythrovirus. J Virol 80: 3666-3669.

79. Parsyan A, Szmaragd C, Allain JP, Candotti D (2007) Identification and genetic diversity of two human parvovirus B19 genotype 3 subtypes. J Gen Virol 88: 428-431.

80. Robles-Sikisaka R, Bohonak AJ, McClenaghan LR, Jr., Dhar AK (2010) Genetic signature of rapid IHHNV (infectious hypodermal and hematopoietic necrosis virus) expansion in wild Penaeus shrimp populations. PLoS ONE 5: e11799.

81. Streck AF et al. (2011) High rate of viral evolution in the capsid protein of porcine parvovirus. J Gen Virol.

82. Firth C et al. (2010) using time-structured data to estimate evolutionary rates of double-stranded DNA viruses. Mol Biol Evol 27: 2038-2051.

83. Shackelton LA, Rambaut A, Pybus OG, Holmes EC (2006) JC virus evolution and its association with human populations. J Virol 80: 9928-9933.

84. Babkin IV, Babkina IN (2011) Molecular dating in the evolution of vertebrate poxviruses. Intervirology 54: 253-260.

85. Carrasco P, de la Iglesia F, Elena SF (2007) Distribution of fitness and virulence effects caused by single-nucleotide substitutions in Tobacco Etch virus. J Virol 81: 12979-12984.

86. Domingo-Calap P, Cuevas JM, Sanjuán R (2009) The fitness effects of random mutations in single-stranded DNA and RNA bacteriophages. PLoS Genet 5: e1000742.

87. Sanjuán R, Moya A, Elena SF (2004) The distribution of fitness effects caused by single-nucleotide substitutions in an RNA virus. Proc Natl Acad Sci USA 101: 8396-8401.

88. Sanjuán R (2010) Mutational fitness effects in RNA and ssDNA viruses: common patterns revealed by site-directed mutagenesis studies. Phil Trans R Soc Lond 365: 1975-1982.
